# Parasitic Infections of Bicoloured White-toothed Shrew (*Crocidura leucodon*) from Dasht-e-Razan, Western Iran

**Published:** 2017

**Authors:** Ali YOUSEFI, Ali ESLAMI, Sadegh RAHBARI, Iraj MOBEDI

**Affiliations:** 1. Young Researchers and Elites Club, Science and Research Branch, Islamic Azad University, Tehran, Iran; 2. Dept. of Microbiology, Science and Research Branch, Islamic Azad University, Tehran, Iran; 3. Dept. of Medical Parasitology and Mycology, School of Public Health, Tehran University of Medical Sciences, Tehran, Iran

**Keywords:** Parasite, Shrew, *Crocidura leucodon*, Iran

## Abstract

**Background:**

The prevalence and intensity of endo and ectoparasites in shrews inhabiting in the Dasht-e Razan of Hamedan Province, Iran, were determined in this study.

**Methods:**

By live traps, 64 shrews belong to species bicoloured white-toothed shrews (*Crocidura leucodon*) were trapped during 2010–2012. Captured animals were euthanized and their gender recorded. The blood thick and thin smears were stained with Geimsa and examined for protozoan parasites. Then, ectoparasites were collected and preserved in 70% ethanol and after necropsies; different organs were examined for helminthes.

**Results:**

The prevalence of collected helminthes of *Crocidura leucodon* were; *Capillaria crociduri* (18.7%)*, Vigisolepis secunda* (26.5%), *Coronacantus* sp (15.6%), *Capillaria hokkaidensis* (45.3%)**,** and its ectoparasites were; Nymphs of three species of ticks; *Haemaphysalis* sp (32.8%), *Ornitodoros* sp (23.4%), *Hyalomma* sp (9.4%), one species of louse, *Polyplax reclinata* (18.7%) and one species of flea *Leptopsylla* sp (39.1%). Among the collected parasites, all helminthes and one sucking louse, *P. reclinata* are reported for the first time in Iran. Statistically analysis with the Chi-square test did not show any significant relation between gender and endoparasites (*P*>0.05), but the ectoparasites had significant differences with gender (*P*<0.05). No significant correlation was found between the altitude and the parasite species richness (Spearman’s test: *P*>0.05).

**Conclusion:**

This study reports 9 species of parasites and 5 species of them were identified for the first time in Iran and some of them are vectors of several important zoonoses agents.

## Introduction

Shrews, including *Crocidura leucodon* in this study are among the smallest mammals in the world (1). Their phenotype is apparently similar to mice and lives in vary widely terrestrial habitats, from mountain or boreal regions to arid areas and some of them are accustomed to living in humans and houses (2).

They are insectivores and feed of invertebrates and insects (3), but occasionally feed on stored foods and contaminate them with feces and urine (4), hence, potential exists for the transmission of pathogens like Hanta virus (5–6), Ebola virus (7) and *Toxoplasma gondii* (8). Moreover, they can play an important role as reservoir hosts for vector borne diseases agents, such as *Brugia* spp (9) and *Angiostrongylus cantonensis* (10) and harbored for the ectoparasites, which may be attack humans and transmit the infections or causing allergic diseases.

Several studies have been documented in parasites of shrews in the different countries, such as Finland (11), Spain (12), Lithuania (13), Hungary (14) and Czech (15).

The objectives of this research were to determinate prevalence and intensity of endo and ectoparasites in *C. leucodon* from Dasht-e Razan in Hamedan Province, for the first time in Iran.

## Materials and Methods

### Study area

**Fig. 1:**
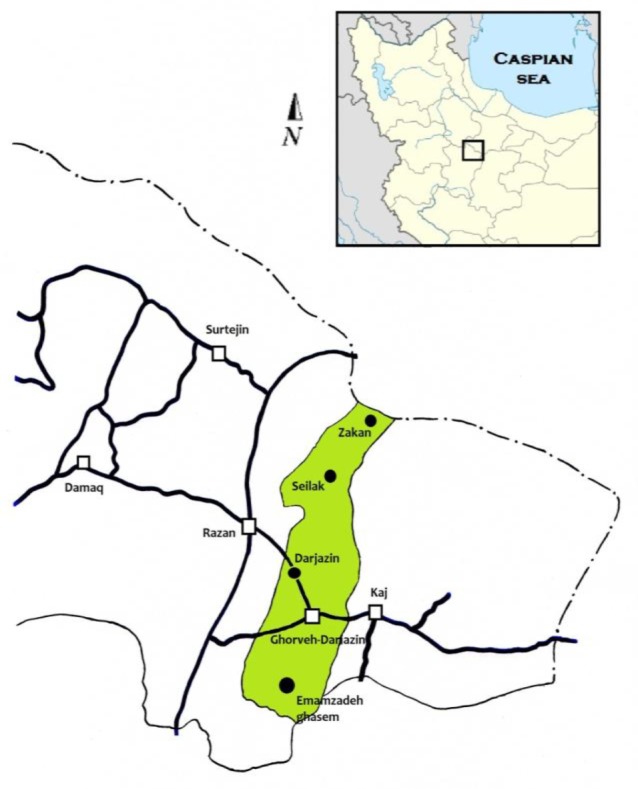
Map of studied areas, showing the Dasht-e Razan

Dasht-e Razan is situated in the north of Hamedan province (Fig. 1) and in the east part of Zagros Mountain. The annual average precipitation and temperature are 300 mm and 11 °C, respectively.

### Collection of shrews and Methods

We conducted fieldwork from Feb 2010 to Nov 2012 at four-study site in the Dasht-e Razan. These sites included Darjazin, Zakan, Seilak, Emamzadeh-Ghasem (Table 1), where 64 shrews of 1 species (*C. leucodon*) were collected by live traps (Fig. 1).

**Table 1:** Localities detail of capture sites of *Crocidura leucodon* in the present study (Fig. 1)

**Locality**	**Altitude (m.a.s.l.)**	**Coordinates**	**N**
Darjazin	1821	35°21′ N 49°04′ E	23
Zakan	2216	35°27′ N 49°09′ E	10
Seilak	1991	35°26′ N 49°07′ E	17
Emamzadeh-Ghasem	1793	35°18′ N 49°02′ E	14

N- number of hosts; M.A.S.L- meters above sea level

The captured animals were anesthetized and after recorded of gender, they were autopsied with using standard animal ethics. Their skulls were limbed and heads were sent to Department of Biology of Tehran University and shrews identified with aid of references book (1, 2, 16).

The blood was taken using the heart puncture method, blood smear prepared and stained by the standard Giemsa’s method and the slides were examined for protozoan parasites under a light microscope.

Ectoparasites collected by skin surface brushing into ethanol (70%) containers in the field. Subsequently in laboratory, they were cleared in 10% aqueous potassium hydroxide, dehydrated and mounted with Canada balsam for more investigation.

After dissection, different organs, including: pectoral area, abdominal cavity, liver, lungs, esophagus, stomach, small intestine, large intestine and bladder were examined for helminthes. Muscles were digested in the acid and pepsin for 12 h at 37 °C in incubator for collected the larvae. Nematodes collected in 5% formalin solution and placed under a drop of lacto phenol on temporary mount. Tapeworms were allowed to relax in tap water, fixed in AFA solution, stained by acetocarmine and cleared in xylol for identification (17–22).

### Statistical analysis

Data management was performed using SPSS ver. 21.0 (Chicago, IL, USA). The Chi-square test was used to determine the relation between gender and parasites (statistically significant differences were determined at *P*<0.05.). Spearman’s test was performed to determining the correlation between altitude of the sampled points and parasite richness.

## Results

Results of helminthes infections of 64 *C. leucodon* (29 males, 35 females) are summarized in Table 2. Among the different organs, digestive tracts and bladders harbored three and one species of helminthes respectively.

**Table 2:** Prevalence and intensity of helminthes in 64 *Crocidura leucodon* from Dasht-e Razan, Hamedan

**Parasites**	**Male (n=29)**			**Female (n=35)**			**Total**		
**(Organ)**	**P%**	**MI**	**RI**	**P%**	**MI**	**RI**	**P%**	**MI**	**RI**
*Capillaria crociduri* (Stomach)	18.7	1.9	1–5	25.7	2.6	1–5	10.3	1.7	1–3
*Vigisolepis secunda* (Small intestine)	26.5	7.2	3–27	34.3	5.3	3–14	17.2	11.6	3–27
*Coronacantus sp* (Small intestine)	15.6	3.1	2–6	14.3	3.4	2–6	17.2	2.8	2–4
*Capillaria hokkaidensis* **(**Urinary bladder**)**	45.3	12.4	6–21	48.6	11.2	6–16	41.4	14	11–21

P% - prevalence, MI- mean intensity, RI- range of the intensity

The data in Table 2 would be revealed that all the identified helminthes are reported for the first time in Iran and the total range of prevalences were medium (15.6%–45.3%) and those of intensities were low (1.9%–12.4%).

**Fig. 2:**
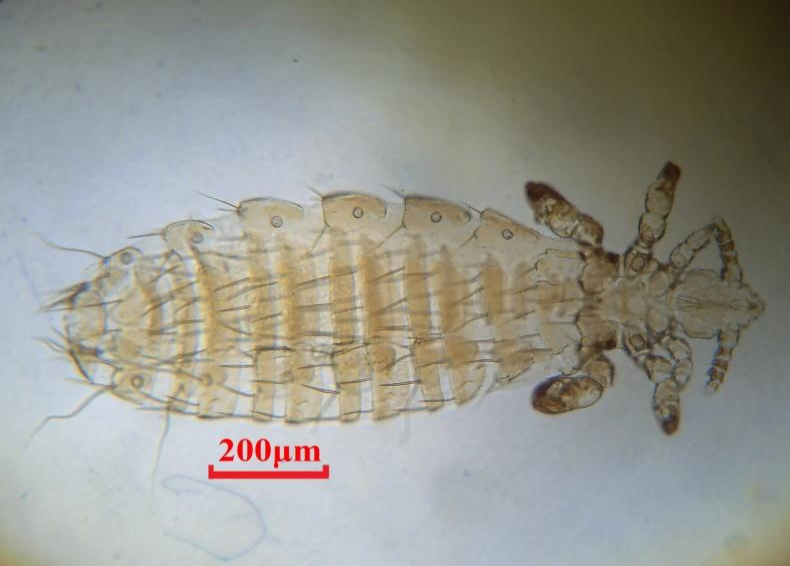
*Polyplax reclinata*, adult female (Original)

In the results of ectoparasites investigation, five species (1 sucking louse, 1 flea and 3 nymphs of ticks) were recovered from *C. leucodon* and the results of them were summarized in Table 3. According to Table 3, *Leptopsylla* sp (39.1%), and *Hyalomma* sp (9.4%) were the most, and least prevalence, respectively. The highest intensity was observed at *P. reclinata* (67), moreover *P. reclinata* (Fig. 2) recorded for the first time in Iran. Total prevalence of endo and ectoparasites in *C. leucodon* according to the gender showed in Table 4.

**Table 3:** Prevalence and intensity of ectoparasites in *Crocidura leucodon* from Dasht-e Razan, Hamedan

**Ectoparasite species**	**Male (n=29)**			**Female (n=35)**			**Total**		
**`**	**P%**	**MI**	**RI**	**P%**	**MI**	**RI**	**P%**	**MI**	**RI**
**Louse** *Polyplax reclinata*	11.8	17.2	7–25	20	19	7–67	18.7	16	7–67
**Flea** *Leptopsylla* sp	1.8	37.9	1–3	40	1.9	1–5	39.1	1.9	1–5
**Nymphs of ticks** *Haemaphysalis* sp	1.4	48.3	1–2	20	2.9	1–4	32.8	1.9	1–4
*Ornitodoros* sp	4.4	17.2	2–8	28.6	3.8	2–6	23.4	4	2–8
*Hyalomma* sp	2.5	13.8	1–3	5.7	1.5	1–2	9.4	2.2	1–3

P% - prevalence, MI- mean intensity, RI- range of the intensity

**Table 4:** The prevalence of parasites according to the gender in captured shrews from Dasht-e Razan, Hamedan

**Infections**	% **Infection**	% **Infection in genders**	
	`	**Male**	**Female**
Endoparasites	68.75	62.06	74.28
Ectoparasites	71.87	86.21	60

The statistical analysis did not show any significant relation between gender and endoparasites (*P*>0.05). However, significance was observed between gender and ectoparasites (*P*<0.05). No significant correlation between the altitude and the parasite species richness was found (Spearman’s test: *P*>0.05). Among this study did not find any tissue or blood parasites.

## Discussion

There are seven species of *Crocidura* in Iran (23) and among them, one species (*C. leucodon*) was collected from Dasht-e Razan in Hamedan province and its parasite infections studied for the first time in Dasht-e Razan as well as in Iran.

**Fig. 3:**
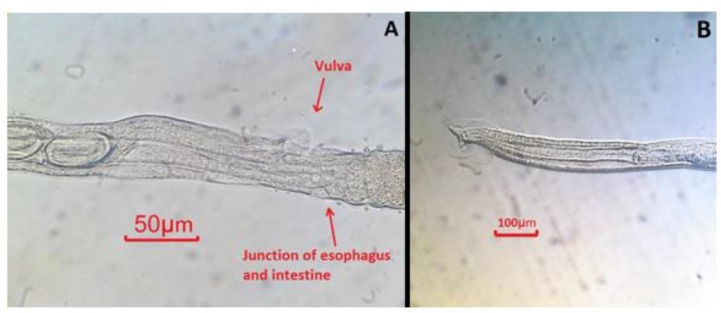
*Capillaria crociduri* (Original) **A:** Vulva, egg, and junction of esophagus and intestine of female, right-lateral view, **B:** Anterior extremity of spicule of male

**Fig. 4:**
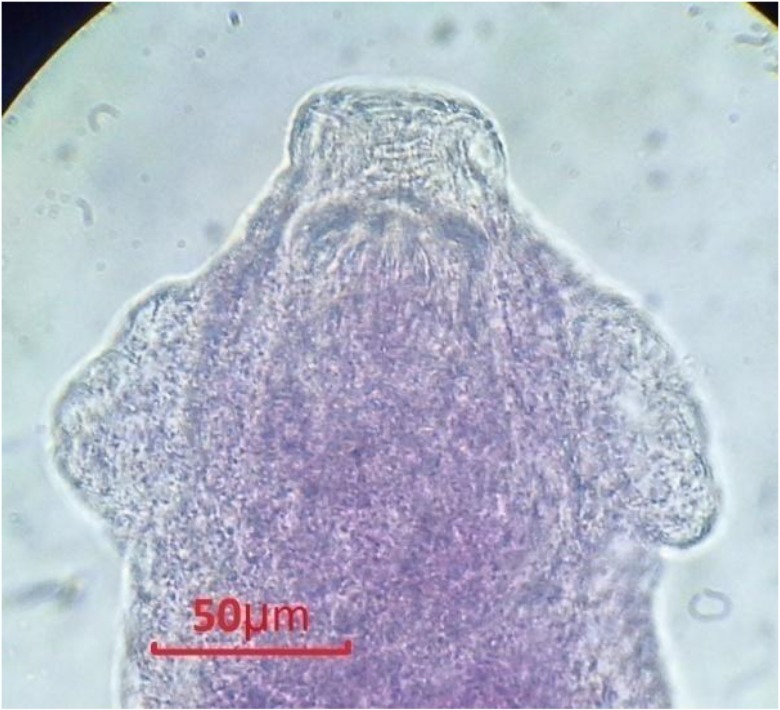
*Vigisolepis secunda*, Scolex (Original)

**Fig. 5:**
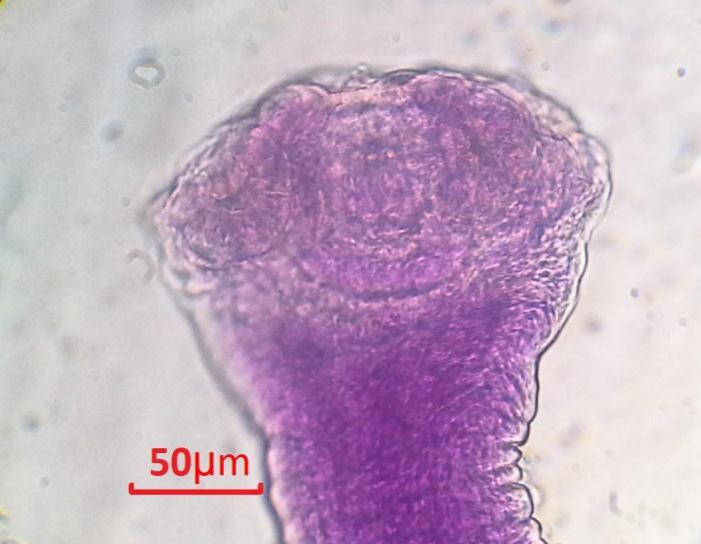
*Coronacantus* sp, Scolex (Original)

**Fig. 6:**
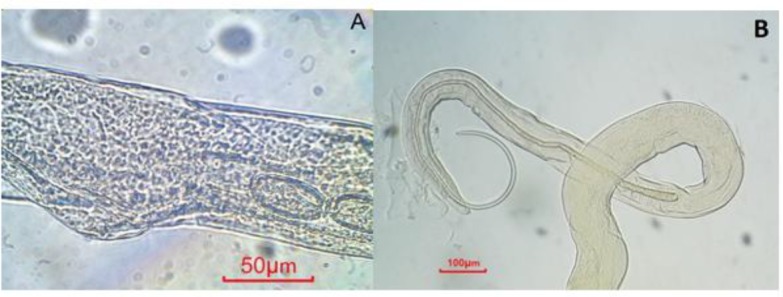
*Capillaria hokkaidensis* (Original) **A:** Vulva and egg of female, left-lateral view, **B:** Anterior extremity of spicule of male

Generally *C. leucodon* (n=64) were infected with 4 species of helminthes and 5 species of ectoparasites which among the collected parasites, namely: *C. crociduri* (Fig. 3)*, V. secunda* (Fig. 4)*, Coronacantus sp* (Fig. 5)*, C. hokkaidensis* (Fig. 6) and *P. reclinata* all identified for the first time in Iran. Meanwhile, *C. hokkaidensis* was recovered from new host previously reported in different species of *Sorex* (19).

There are very scarce references on the helminthes of *C. leucodon* in the world, such as; Lake Balaton (14) and Ferto-Hansag National Park (24) which in them respectively 3 (of twelve checked) and 2 (of nineteen checked) helminthes reported which are differ from the present findings. In the helminthe infections from the japanese shrews (19) out of 5 helminthes reported and among them, *C.crociduri* and *C.hokkaidensis* were similar to our results. The prevalence of helminthes is medium (45.3%–15.6%) but their frequencies low (12.4%–1.9%) which corresponds to other investigations results on shrews (14–15).

The ectoparasites are more important than endoparasites, because small mammal like shrew and rodents are widely distributed in the present study area and they are harboring a number of agents that could be transmitted diseases to human and domestic animals in densely populated urban and rural areas.

In this investigation, 5 species of ectoparasites were observed on *C. leucodon* (Table 3), all except that of *P. reclinata* were previously reported from Iran. Statistical analysis was show significant differences between gender and ectoparasites (*P*<0.05).

In researches on ectoparasites e.g. parasitic alien terrestrial arthropods on small mammals in Northeast and South Bulgaria (25), mammal density and patterns of ectoparasite species richness and abundance (26), respectively 3 and 5 species of ectoparasites were similar to our results.

Already, findings on Siphonaptera were reported three species including; *Leptopsylla segnis, L. aethiopicus aethiopicus* and *L. taschenbergi taschenbergi* from Iran (27–29). The species of *Leptopsylla* are vectors of several important zoonoses agents, such as *Yersinia pestis, Rickettsia typhi* and *Rickettsia mooseri* (30–32).

Ticks are vectors of important pathogens for humans and animals and serve as indicators of infection in nature (33) such as tick-borne relapsing fever and Q fever being respectively, transmitted by *Ornithodoros* spp and *Hyalomma* spp (34).

One of principal vector of tularemia and tick-borne encephalitis is Haemaphysalis concinna (35) that nymphs and larvae feed on small mammals (36). In addition, Haemaphysalis and Hyalomma genera could have important role in spread of Crimean–Congo hemorrhagic fever (CCHF) that is a zoonosis of domestic animals and wild animals, which may affect humans (37).

## Conclusion

Endo and ectoparasites infestation rate is high (68.75%–71.87%) and some of them are vectors of several important zoonoses agents. Therefore, shrews have a role in spread of infectious agents in environment. Thus, further parasitological studies are required due to noticeable in other areas of Iran in order to ascend our knowledge to verify the species of parasites of these small mammals and probable zoonoses and veterinary diseases.
